# Cell‐free lncRNA expression signatures in urine serve as novel non‐invasive biomarkers for diagnosis and recurrence prediction of bladder cancer

**DOI:** 10.1111/jcmm.13578

**Published:** 2018-03-08

**Authors:** Lutao Du, Weili Duan, Xiumei Jiang, Li Zhao, Juan Li, Rui Wang, Suzhen Yan, Yujiao Xie, Keqiang Yan, Qingliang Wang, Lili Wang, Yongmei Yang, Chuanxin Wang

**Affiliations:** ^1^ Department of Clinical Laboratory The Second Hospital of Shandong University Jinan Shandong China; ^2^ Tumor Marker Detection Engineering Laboratory of Shandong Province Jinan Shandong China; ^3^ Department of Clinical Laboratory Tai'an Tumour Prevention and Treatment Hospital Taian Shandong China; ^4^ Department of Urology Qilu Hospital of Shandong University Jinan Shandong China; ^5^ Department of Clinical Laboratory Qilu Hospital of Shandong University Jinan Shandong China

**Keywords:** bladder cancer, diagnosis, lncRNA, prognosis, urine

## Abstract

Cell‐free long non‐coding RNAs (lncRNAs) are stably present in urine and can serve as non‐invasive biomarkers for cancer. We aimed to identify signatures of lncRNAs in urine for diagnosis and prognosis of bladder cancer (BC). Screening of lncRNAs by microarray analysis was performed using urine samples of 10 BC patients and 10 controls. Expressions of candidate lncRNAs were evaluated in the training and validation set including 230 BC patients and 230 controls by quantitative reverse transcription polymerase chain reaction (qRT‐PCR). A two‐lncRNA panel (uc004cox.4 and GAS5) was constructed and provided high diagnostic accuracy of BC with an area under the curve (AUC) of 0.885 (95% CI, 0.836‐0.924). The AUCs of the lncRNA panel for Ta, T1 and T2‐T4 were 0.843, 0.867 and 0.923, respectively, significantly higher than those of urine cytology (all *P* < .05). Kaplan‐Meier analysis revealed that higher level of uc004cox.4 was associated with poor recurrence‐free survival (RFS) of non‐muscle invasive BC (NMIBC) (*P* = .008). Additionally, Cox regression analysis indicated that uc004cox.4 was an independent prognostic factor for RFS of NMIBC (*P* = .018). Taken together, our findings indicated that urinary lncRNA signatures possessed potential clinical value for BC diagnosis. Moreover, uc004cox.4 could provide prognostic information for NMIBC.

## 1 INTRODUCTION

Bladder cancer (BC) is one of the most common urological malignancies worldwide, with estimated 79 030 new cases and 16 870 BC‐related deaths in 2017 in the United States.^1^ Early diagnosis and treatment of cancerous or precancerous lesions is thought to be critical important for reducing the risk of relapse and improving the prognosis of BC. Currently, voided urine cytology, as the most widely used non‐invasive approach, has high specificity for detection and monitoring of BC. However, this test has limited diagnostic sensitivity, which is necessary to rule out cancer.^2^, ^3^ The histological evaluation of cystoscopy‐guided biopsy can provide high diagnostic accuracy, but it is invasive and often inconvenient, making it impractical for mass screening of BC in people without signs or symptoms of disease. These characteristics have prompted a search of novel biomarkers, particularly more reliable non‐invasive markers for screening, initial evaluation and follow‐up of BC.

As a new class of non‐coding RNAs, long non‐coding RNAs (lncRNAs) are more than 200 nucleotides in length with no or lower protein‐coding potential. Moreover, lncRNAs have been continually reported to be involved in tumorigenesis and progress.^4^, ^5^, ^6^ Dysregulated lncRNA expression has been found in many human malignancies.^7^, ^8^ Extracellular lncRNAs can circulate in body fluids and can be detected and strongly resist RNases. Several studies have investigated the potential of using lncRNAs as biomarkers for cancer diagnosis, and promising results have been obtained. In those studies, some lncRNAs were readily detected and remained stable in whole blood, plasma, gastric juice and saliva, which suggested that lncRNAs could be good candidates for tumour biomarkers.^9^ Researchers have also found that lncRNAs in urine are detectable and may serve as new predictors of outcome in kidney‐transplant patients with acute rejection.^10^ However, detection of lncRNAs in urine of BC patients has not been investigated yet. Differentially expressed lncRNAs have already been identified between BC tissues and normal tissues.^11^, ^12^ The dysregulated expression of lncRNAs may imply their potential application as biomarkers for BC screening.

In this study, we firstly performed a microarray analysis to identify the genome‐wide different expression profiles of lncRNAs between BC and control urine specimens. Then we selected 26 candidate lncRNAs and evaluated their expression levels by quantitative reverse transcription polymerase chain reaction (qRT‐PCR) in the training set and validation set. Finally, we established a two‐lncRNA panel with high diagnostic accuracy for BC. In addition, we further investigated the potential relationship between urinary lncRNA levels and recurrence of BC.

## 2 MATERIALS AND METHODS

### 2.1 Study design

A total of 240 BC patients and 240 controls were recruited from Qilu Hospital, Shandong University, between February 2008 and March 2011. All the participants were randomly divided into three phases. In the discovery set, pooled urine samples from 10 BC patients and 10 healthy controls were, respectively, subjected to microarray assay to initially identify dysregulated lncRNAs in BC. In the training set, the expression levels of candidate lncRNAs were firstly investigated in an independent cohort containing 36 BC patients and 36 controls. LncRNAs that were differentially expressed between BC group and control group were further examined in an additional test consisting of 168 participants including 84 BC patients and 84 controls. These above‐mentioned 240 urine samples were used as the training set to construct the diagnostic panel based on the logistic regression model to distinguish BC patients from controls. In the validation set, the parameters of the logistic model from the training set were applied to an independent cohort consisting of 110 BC patients and 110 controls to confirm the diagnostic performance of the established lncRNA panel. Additionally, urine cytology was also performed on the same cohort to further assess the diagnostic value of the constructed lncRNA panel. Meanwhile, BC patients in the validation set were followed up at intervals of 3 months during the first 2 years and 6 months up to the fifth year. The date of the latest retrieved record was April 2016.

### 2.2 Patients and control subjects

This study was approved by the Clinical Research Ethics Committee of Qilu Hospital, Shandong University, and informed consent was received from every participant. Urine samples were obtained before undergoing transurethral resection (TUR) or radical cystectomy. Tumour stage of BC was determined following the 2002 UICC TNM classification of BC, and tumour grade was identified according to the WHO 2004 grading scheme. Table S1 summarizes the demographic and clinical features of participants.

### 2.3 Urine sample preparation

Freshly voided urine samples were collected on the day before treatment. Cells and debris were removed from each urine sample by centrifugation at 1500× *g* for 10 minutes and 13 800× g for 15 minutes at 4°C, and supernatant was stored at −80°C prior to further analysis. In addition, 15‐mL mid‐stream urine of BC patients was centrifuged at 1300× *g* for 10 minutes, and sediments were immediately processed for cytologic examination by two independent cytopathologists.

### 2.4 Microarray assay

Total RNA was extracted from urine samples of BC patients and healthy controls using Trizol LS Reagent (Invitrogen Life Technologies, Carlsbad, USA). Total RNA was amplified and transcribed into fluorescent cRNA along the entire length of the transcripts without bias utilizing a random priming method. cRNA was labelled and hybridized to human lncRNAs Array v2.0. The microarray hybridization and collection of expression data were performed by KangChen Bio‐Tech, Shanghai, China. After washing the slides, the arrays were scanned by the Agilent DNA Microarray Scanner. Agilent Feature Extraction software (version 10.7.3.1) was used to analyse the acquired array images. Raw signal intensities were normalized by quantile method through GeneSpring GX v 11.5.1.

### 2.5 RNA isolation and qRT‐PCR

Total RNA was isolated from 400 μL urine with the miRNeasy Mini Kit (Qiagen) according to the manufacturer's instructions. NanoDrop spectrophotometer was used to measure the concentration of isolated RNA. Reverse transcription of urinary lncRNA was performed using the PrimeScript™ RT reagent kit (Takara, Dalian, Liaoning). The 20 μL RT volume consists of 1 μg of template RNA, 4 μL of 5 × PrimeScript Buffer, 1 μL of PrimeScript RT Enzyme Mix I, 1 μL of Oligo dT Primer and RNase‐free dH_2_O. The mixture was briefly centrifuged and incubated at 37°C for 30 minutes, followed by incubation at 85°C for 5 seconds and 4°C for 60 minutes. The quantitative polymerase chain reaction was conducted in a 25‐μL reaction system containing 12.5 μL of SYBR^®^ Premix Ex Taq™, 0.5 μL of ROX Reference Dye α, 1 μL of forward primer (10 μmol/L), 1 μL of reverse primer, 8 μL of RNase‐free dH_2_O and 2 μL of cDNA on a CFX‐96 real‐time PCR System using the SYBR^®^ Premix Ex Taq™ (Takara, Dalian, Liaoning). The mixture was incubated at 95°C for 30 seconds, followed by 42 cycles at 95°C for 5 seconds and 60°C for 34 seconds. All reactions were performed in triplicate. Specificity of qRT‐PCR product was measured by melting curve analysis. GAPDH was selected as the housekeeping gene, and the relative expressions of lncRNAs were calculated using the 2^−▵▵ct^ method.

### 2.6 Statistical analysis

Statistical analyses were performed using SPSS software (version 19.0, SPSS, Chicago, IL, USA). Nonparametric Mann‐Whitney U‐tests were employed to compare differences in the urinary expression levels of lncRNAs between BC patients and controls. Receiver operating characteristic (ROC) curve was established to discriminate BC patients from controls using MedCala 9.3.9.0 (MedCala, Mariakerke, Belgium). Area under the ROC curve (AUC) was used to evaluate the diagnostic performance. The Youden index (sensitivity + specificity − 1) was used to set the optimal cut‐off point.^13^ Survival curves were established by Kaplan‐Meier method, and differences were assessed using log‐rank statistics. Univariate and multivariate Cox analyses were employed to identify independent prognostic factors of BC. A *P*‐value < .05 was considered as statistically significant.

## 3 RESULTS

### 3.1 Global screening of lncRNA expression in BC by microarray

To identify dysregulated lncRNAs in BC, we assessed the lncRNA expression profiles in urine samples of 10 BC patients and 10 controls by microarray (GSE106074). A total of 3093 lncRNAs were differentially expressed (fold change ≥ 2.0) between BC patients and controls. Among them, 1680 lncRNAs were up‐regulated and 1413 lncRNAs were down‐regulated in BC patients. Of all the differentially expressed lncRNAs indicated by lncRNA expression profiling, 26 lncRNAs (fold change>25) were firstly selected for further evaluation, including 16 up‐regulated lncRNAs and 10 down‐regulated lncRNAs in BC patients.

### 3.2 Evaluation of lncRNA expression by qRT‐PCR in training set and validation set

**Figure 1:**
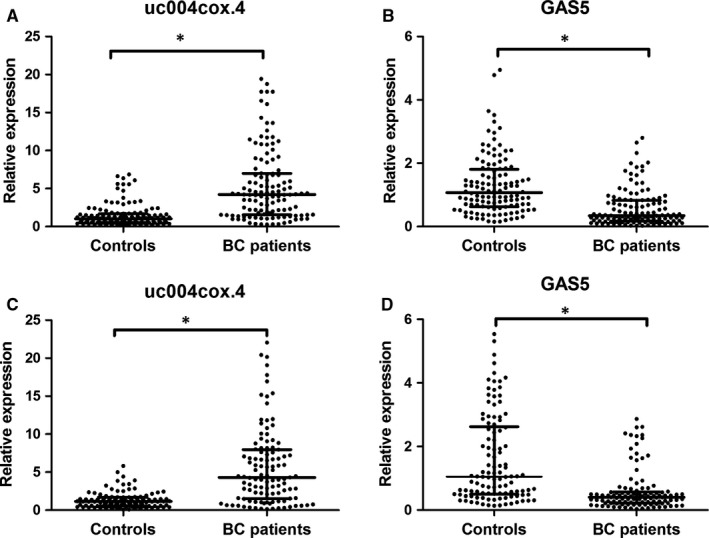
Expression levels of urinary uc004cox.4 and GAS5 in BC patients and controls by qRT‐PCR. Expression levels of uc004cox.4 in the training set (A), GAS5 in the training set (B), uc004cox.4 in the validation set (C) and GAS5 in the validation set (D)

**Figure 2:**
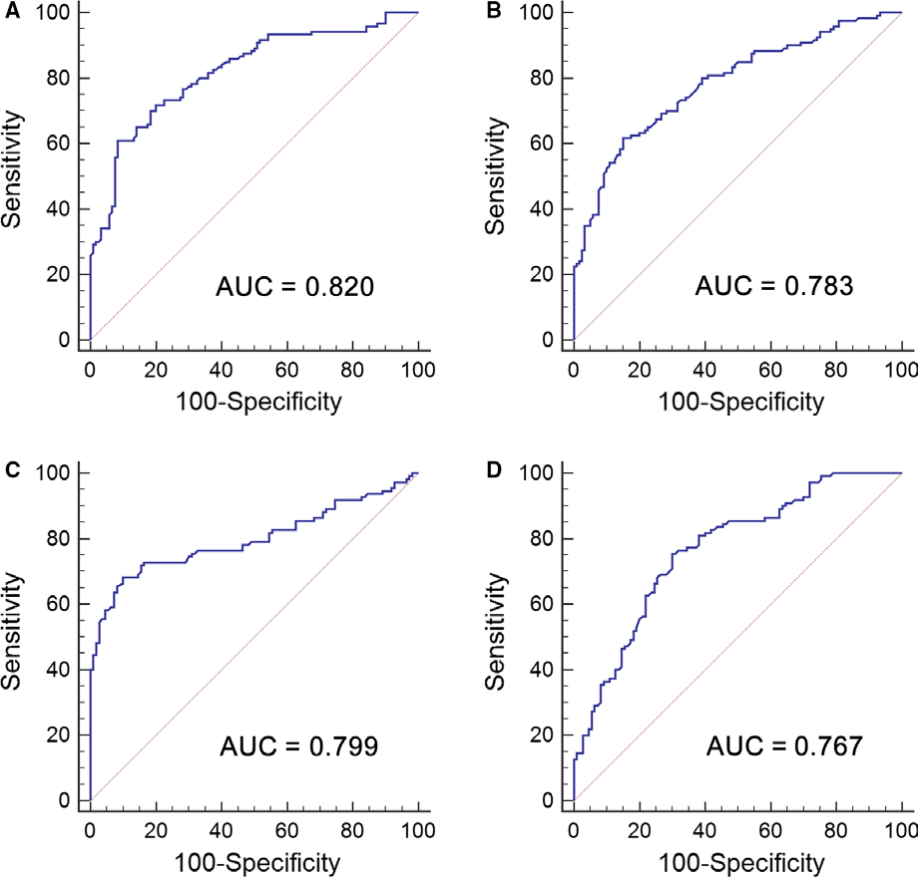
Diagnostic performance of deregulated lncRNAs in urine for BC. ROC curve analysis for BC detection using uc004cox.4 (A) and GAS5 (B) in the training set, or uc004cox.4 (C) and GAS5 (D) in the validation set

The 26 candidate lncRNAs revealed in microarray were firstly investigated by qRT‐PCR assays in an independent cohort consisting of 36 BC patients and 36 controls. Only differently expressed lncRNAs were selected for further validation. We determined that the expressions of uc004cox.4 and ENST00000414075 (GAS5) were significantly changed between BC patients and controls (*P* < .05). The expressions of uc004cox.4 and GAS5 were then evaluated in another cohort consisting of 84 BC patients and 84 controls. These above‐mentioned 120 BC patients and 120 controls were used as the training set. In the training set, uc004cox.4 was up‐regulated and GAS5 was down‐regulated in BC patients (Figure. 1A,B). ROC analysis revealed that the AUC of uc004cox.4 and GAS5 for BC diagnosis was 0.820 (95% CI, 0.765‐0.866) and 0.783 (95% CI, 0.726‐0.834), respectively (Figure 2A,B).

The expressions of uc004cox.4 and GAS5 were then assessed in the validation set, including 110 BC patients and 110 controls. The results obtained from the validation set were consistent with the training set (Table 1). In the validation set, the uc004cox.4 was significantly up‐regulated and GAS5 was down‐regulated in BC patients (Figure 1C,D). The AUC of uc004cox.4 and GAS5 was 0.799 (95% CI, 0.740‐0.850) and 0.767 (95% CI, 0.706‐0.821), respectively (Figure 2C,D).

**Table 1:** The selected urinary lncRNA concentrations in BC patients and control individuals in training set and validation set [median (interquartile range)]

lncRNA	Training set			Validation set		
	Controls (n = 120)	BCs (n = 120)	*P*‐Value	Controls (n = 110)	BCs (n = 110)	*P*‐Value
Uc004cox.4	1.02 (0.54‐1.70)	4.21 (1.56‐7.00)	<.001	1.15 (0.55‐1.67)	4.30 (1.52‐7.98)	<.001
GAS5	1.07 (0.63‐1.81)	0.34 (0.18‐0.83)	<.001	1.05 (0.50‐2.62)	0.40 (0.22‐0.57)	<.001

### 3.3 Establishment of urinary lncRNA panel for BC diagnosis by logistic regression model analysis

**Figure 3:**
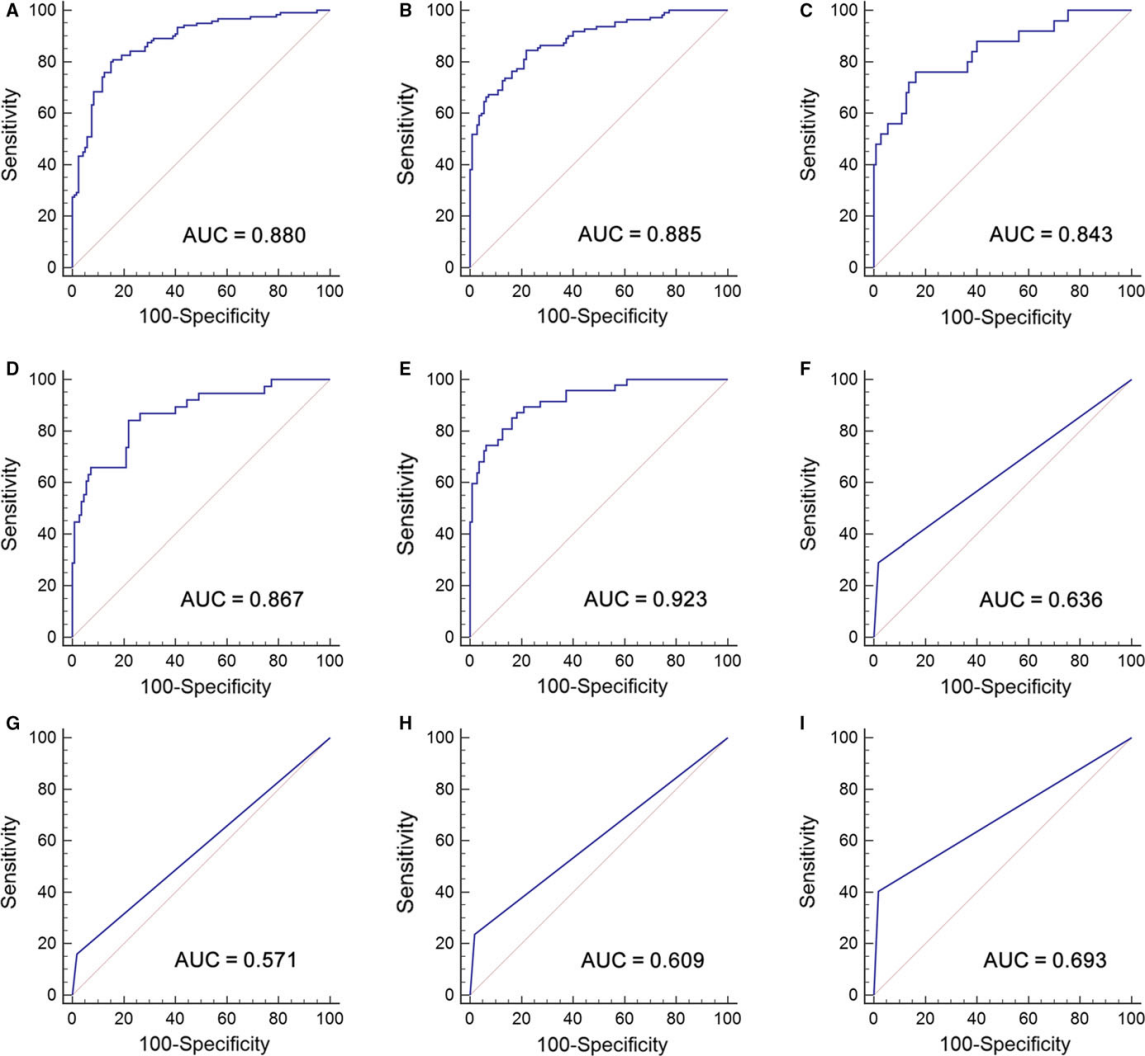
Diagnostic performance of two‐lncRNA panel and urine cytology for BC detection. ROC analysis using two‐lncRNA panel for BC detection in the training set (A) and in the validation set (B); ROC curves showing the diagnostic performance of the two‐lncRNA panel for Ta (C), T1 (D) and T2‐T4 (E) in the validation set; ROC curve analysis using urine cytology for BC detection with all stages (F), Ta (G), T1 (H) and T2‐T4 (I) in the validation set

A stepwise logistic regression model was employed to estimate the risk of being diagnosed with BC in the training set. The predicted probability of being diagnosed with BC from the logit model based on the lncRNA panel, logit (*P*=BC) = 0.0012 − (0.1160 × uc004cox.4) + (0.4227 × GAS5) was used to construct the ROC curve. The diagnostic performance of the established lncRNA panel was evaluated by ROC analysis. The AUC of the established lncRNA panel was 0.880 (95% CI, 0.832‐0.918) with a sensitivity of 80.0% and a specificity of 85.0% (Figure 3A).

### 3.4 Validation of the diagnostic value of the constructed lncRNA panel

The parameters established from the training set were used to predict the probability of being diagnosed with BC in the independent cohort consisting of 220 participants. Similarly, the predicted probability was used to construct the ROC curve. The AUC of the lncRNA panel was 0.885 (95% CI, 0.836‐0.924) with a sensitivity of 84.5% and a specificity of 78.2% in the validation set (Figure 3B). The corresponding AUC of the lncRNA panel for BC patients with stages Ta, T1 and T2‐4 was 0.843 (95% CI, 0.770‐0.900), 0.867 (95% CI, 0.801‐0.917) and 0.923 (95% CI, 0.869‐0.959), respectively, (Figure 3C‐E). The diagnostic accuracy of urine cytology for BC was further evaluated in the same cohort. The AUC of urine cytology to differentiate BC patients from controls was 0.636 (95% CI, 0.569‐0.700, sensitivity = 29.1% and specificity = 98.2%, Figure 3F). The AUCs of the lncRNA panel for BC patients with stages Ta, T1 and T2‐4 were significantly higher than those of urine cytology, which were 0.571 (95% CI, 0.483‐0.656), 0.609 (95% CI, 0.526‐0.688) and 0.693 (95% CI, 0.615‐0.764), respectively (Figure 3G‐I).

### 3.5 Correlation between two lncRNAs and clinicopathological characteristics

Table S2 summarizes the relationship between the two lncRNAs and clinicopathological characteristics of BC patients in the validation set. Higher urinary level of uc004cox.4 was significantly correlated with advanced tumour stage (*P* < .05). However, no significant associations were found between the two lncRNAs and age, sex, tumour grade and lymph node metastasis.

### 3.6 Correlation between the two lncRNAs and tumour recurrence in non‐muscle invasive BC (NMIBC) and muscle‐invasive BC (MIBC)

**Figure 4:**
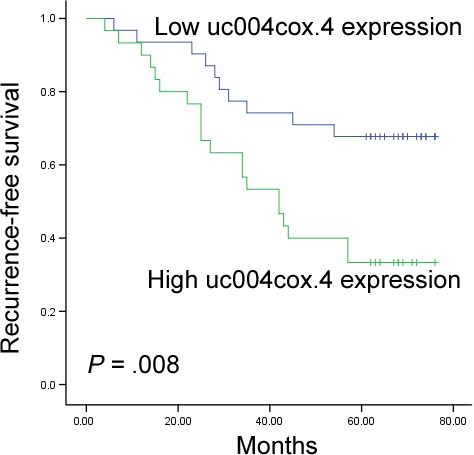
Kaplan‐Meier curves for RFS according to the urinary level of uc004cox.4 in NMIBC patients in the validation set

In the validation set, seven patients, including two NMIBC patients and five MIBC patients, were lost during follow‐up because of incomplete data. Survival analysis was carried in 61 NMIBC and 42 MIBC patients. The median follow‐up time was 61 months (range: 4‐76 months). In the NMIBC group, Kaplan‐Meier analysis showed that higher expression level of uc004cox.4 was associated with poorer recurrence‐free survival (RFS) (*P* = .008) (Figure 4). However, the GAS5 expression level had no correlation with RFS of BC. Subsequently, univariate Cox analysis revealed that the uc004cox.4 expression and tumour stage were significantly correlated with RFS (*P* = .012 and .023, respectively). Furthermore, multivariate Cox analysis indicated that uc004cox.4 and tumour stage maintained their significance as independent prognostic factors for RFS of NMIBC (*P* = .018 and .034, respectively) (Table 2). In the MIBC group, however, none of these two lncRNAs influenced the RFS of patients (Table S3).

**Table 2:** Univariate and multivariate Cox proportional hazards regression model analysis of RFS in patients with NMIBC in validation set

Parameters	Categories	Univariate analysis		Multivariate analysis	
		HR (95%CI)	*P*‐Value	HR (95%CI)	*P*‐Value
Age	≤66 vs >66	1.119 (0.546‐2.293)	.759		
Sex	Male vs female	0.947 (0.406‐2.208)	.899		
Tumour stage	Ta vs T1	2.575 (1.142‐5.806)	.023	2.422 (1.071‐5.476)	.034
Tumour grade	Low vs high	1.003 (0.470‐2.144)	.993		
Lymph node metastasis	Negative vs positive	0.977 (0.232‐4.107)	.975		
uc004cox.4 expression	Low vs high	2.667 (1.245‐5.713)	.012	2.524 (1.175‐5.419)	.018
GAS5 expression	Low vs high	1.295 (0.631‐2.657)	.480		

CI, Confidence interval; HR, Hazard ratio; RFS, Recurrence‐free survival.

## 4 DISCUSSION

Current methods for the BC diagnosis mainly fall into two categories: cystoscopy and urine cytology. However, the performance of cystoscopy for mass cancer screening is limited, and diagnostic value of urine cytology for early‐stage tumours is unsatisfactory. With the aim of overcoming these existing difficulties, several previous studies have been prompted to identify reliable non‐invasive biomarkers for detecting and predicting the biological behaviour of BC. For instance, bladder tumour antigen (BTA), BTA stat, BTA TRAK, nuclear matrix proteins (NMPs), HSPs and cytokeratin have been developed for detection of BC for decades. Although most of these assays have a higher sensitivity compared with urine cytology, they still cannot replace cystoscopy or cytology for diagnosis and follow‐up examination. It has been demonstrated that cell‐free nucleic acids are abundant and detectable in urine of cancer patients, which have great potential as a useful tool for cancer diagnosis.^14^, ^15^ Mengual et al have examined the miRNA expression profiles in urine samples of BC patients and revealed that six urinary miRNAs can be used to distinguish BC patients from controls.^16^ In our previous study, we have performed systematic analysis and identified seven differently expressed urinary miRNAs in BC patients.^17^ However, little is known about the diagnostic utility of urinary lncRNAs in BC.

Abnormal expressions of LncRNAs have been suggested as a major cause of oncogenesis, and various types of cancers can be distinguished because of their unique altered lncRNA signatures.^18^, ^19^, ^20^ Previous studies have confirmed that the expressions of lncRNAs in human body fluids, including serum/plasma and urine, are abundant and quite stable.^9^ Recently, the rapid development of microarray assays makes it possible to efficiently discover aberrantly expressed lncRNAs in cancer. Our laboratory has previously shown that lncRNAs can be detected in serum of BC patients, and serum lncRNAs (MEG3, SNHG16 and MALAT1) have potential values in BC diagnosis.^21^ As urine samples are in direct contact with tumour cells in BC and can be collected easily and non‐invasively, it is an ideal source for the detection of BC that needs constant monitoring. In the present study, the initial screening of urinary lncRNAs was performed by microarray in BC patients and controls, and potential differently expressed lncRNAs were firstly selected for further validation. By two sets of individual qRT‐PCR evaluations, we identified a two‐lncRNA panel (uc004cox.4 and GAS5) with high accuracy for BC diagnosis. Compared with traditional urine cytology, the constructed panel showed higher accuracy for different stages of BC, especially for Ta and T1. To the best of our knowledge, we, for the first time, identified a urinary lncRNA signature in BC by genome‐wide analysis.

Recent studies have focused on the roles of lncRNAs in the development and progression of cancer, paving the way for BC diagnosis with urinary cell‐free lncRNAs. Of the two urinary lncRNAs identified in this study, GAS5 was found to be involved in the carcinogenesis of cancer. As a tumour‐suppressing gene to inhibit malignant proliferation, GAS5 was down‐regulated in tissues of BC, and GAS5 enhancement reduced the chemotherapy resistance to doxorubicin in J82 and T24 cells.^22^ Cao et al revealed that knockdown of GAS5 resulted in an increased percentage of BC cells in S and G2 phase, and a decreased percentage of cells in G1 phase, suggesting that GAS5 was able to suppress cell proliferation in BC.^23^ In addition, GAS5 has been found to be down‐regulated in a wide array of malignant tumours, such as ovarian cancer,^24^ prostate cancer,^25^ non‐small cell lung cancer,^26^ gastric cancer,^27^ hepatocellular cancer 28 and breast cancer,^29^ which can reflect the presence of cancer, staging information, prognosis prediction or treatment choice. Although the expression pattern and possible functional role of lncRNA uc004cox.4 in tumorigenesis of cancer has not been totally elucidated, our work is the first study to report the importance of the uc004cox.4 expression profile, along with GAS5, in association with BC. Future studies are necessary to identify the molecular mechanisms and downstream signalling targets of these circulating lncRNAs in the tumorigenesis and progression of BC.

Previously, several studies have revealed that dysregulation of lncRNAs in tissues of BC was significantly correlated with tumour progression.^30^, ^31^ From a clinical point of view, urinary lncRNAs for BC diagnosis may be also useful in predicting the prognosis of BC patients. In the present study, we identified a clear correlation between urinary uc004cox.4 expression and different stages of BC. Furthermore, we performed Kaplan‐Meier analysis and identified that the increased expression of urinary uc004cox.4 was significantly associated with poorer prognosis in terms of RFS of NMIBC. By univariate and multivariate Cox regression analyses, urinary uc004cox.4 was identified as an independent risk factor for RFS in NMIBC, which notably suggested a major role of uc004cox.4 in prognosis prediction. However, we did not find any dysregulated lncRNAs influencing the RFS of MIBC patients. These results indicated that the urinary level of uc004cox.4 might also serve as a new prognostic biomarker for NMIBC patients, and it could be a useful tool to select patients who are likely to benefit from adjuvant therapy to reduce the risk of recurrence.

Although our results are promising, there are still some limitations. Firstly, we were unable to provide molecular insights into the release mechanisms of uc004cox.4 and GAS5. Raposo et al have suggested that extracellular vesicles, such as exosomes, microvesicles and apoptotic bodies, can contribute to the remarkable stability of cell‐free lncRNAs.^32^ Additional studies are required to describe the origin and protection mechanisms of uc004cox.4 and GAS5 in urine samples of BC patients. Secondly, our study represented a single‐centre research with a relatively small number of BC patients. Larger number of independent cohorts from multi‐centres will be needed to validate our current findings. Thirdly, the specificity of the constructed urinary lncRNA panel for BC diagnosis was not very clear. As this study recruited only BC patients, it would be desirable to include more patients with other types of cancers in urinary tract.

In conclusion, our study identified a two‐lncRNA panel (uc004cox.4 and GAS5) in urine, which could distinguish BC patients from controls with high accuracy. Moreover, the expression of urinary uc004cox.4 was an independent predictor for the recurrence of NMIBC patients. Our findings, if validated in large‐scale study, might be incorporated with future non‐invasive biomarkers for diagnosis and prognosis of BC.

## CONFLICTS OF INTEREST

The authors declare that no potential conflicts of interest were disclosed.
