# Supplementary material for: Cell‐free lncRNA expression signatures in urine serve as novel non‐invasive biomarkers for diagnosis and recurrence prediction of bladder cancer
Source: J Cell Mol Med. 2018 Mar 8;22(5):2838–45. doi: 10.1111/jcmm.13578 (PMC5908122; doi:10.1111/jcmm.13578)
Supplement: Supplementary file 1 [file JCMM-22-2838-s001.docx]

| **Variable** | **Training set** | **Validation set** | ***P*-Value** |
| --- | --- | --- | --- |
|  | **n=240** | **n=220** |  |
| **Control (number)** | 120 | 110 |  |
| **Age (years)** |  |  | 0.70 |
| ＜66 | 58 (48.33%) | 56 (50.91%) |  |
| ≥66 | 62 (51.67%) | 54 (49.09%) |  |
| **Sex** |  |  | 0.32 |
| Male | 96 (80.00%) | 82 (74.55%) |  |
| Female | 24 (20.00%) | 28 (25.45%) |  |
| **Condition** |  |  | 0.98 |
| BPH | 29 (24.17%) | 26 (23.64%) |  |
| Urolithiasis | 15 (12.50%) | 12 (10.91%) |  |
| Cystitis | 18 (15.00%) | 16 (14.55%) |  |
| Healthy | 58 (48.33%) | 56 (50.91%) |  |
| **BC (number)** | 120 | 110 |  |
| **Age (years)** |  |  | 0.59 |
| ＜66 | 61 (50.83%) | 52 (47.27%) |  |
| ≥66 | 59 (49.17%) | 58 (52.73%) |  |
| **Sex** |  |  | 0.91 |
| Male | 92 (76.67%) | 85 (77.27%) |  |
| Female | 28 (23.33%) | 25 (22.73%) |  |
| **Tumor stage** |  |  | 0.18 |
| Ta-T1 | 79 (65.83%) | 63 (57.27%) |  |
| T2-T4 | 41 (34.17%) | 47 (42.73%) |  |
| **Tumor grade** |  |  | 0.21 |
| Low grade | 72 (60.00%) | 57 (51.82%) |  |
| High grade | 48 (40.00%) | 53 (48.18%) |  |
| **Lymph node matastasis** |  |  | 0.51 |
| Negative | 108 (90.00%) | 96 (87.27%) |  |
| Positive | 12 (10.00%) | 14 (12.73%) |  |
| Abbreviations: BPH, benign prostatic hyperplasia; BC, bladder cancer. | | | |

**Supplementary Table S1: Characteristics of study participants in training set and validation set.**
