# Supplementary material for: Cell‐free lncRNA expression signatures in urine serve as novel non‐invasive biomarkers for diagnosis and recurrence prediction of bladder cancer
Source: J Cell Mol Med. 2018 Mar 8;22(5):2838–45. doi: 10.1111/jcmm.13578 (PMC5908122; doi:10.1111/jcmm.13578)
Supplement: Supplementary file 2 [file JCMM-22-2838-s002.docx]

| **Parameters** | **Total cases** | **uc004cox.4** | ***P*** | **GAS5** | ***P*** |
| --- | --- | --- | --- | --- | --- |
| **Age** | | | 0.88 |  | 0.72 |
| ＜66 | 52 | 4.43 (2.12-7.88) |  | 0.40 (0.20-0.66) |  |
| ≥66 | 58 | 4.09 (1.20-8.28) |  | 0.38 (0.21-0.52) |  |
| **Sex** | | | 0.91 |  | 0.20 |
| Male | 85 | 4.12 (1.56-7.99) |  | 0.42 (0.22-0.66) |  |
| Female | 25 | 4.39 (1.21-7.63) |  | 0.31 (0.20-0.46) |  |
| **Tumor stage** | | | 0.02 |  | 0.27 |
| Ta-T1 | 63 | 3.71 (1.06-7.23) |  | 0.41 (0.24-0.58) |  |
| T2-T4 | 47 | 5.95 (2.61-10.08) |  | 0.33 (0.17-0.51) |  |
| **Tumor grade** | | | 0.41 |  | 0.25 |
| Low grade | 57 | 4.01 (1.14-7.48) |  | 0.39 (0.24-0.68) |  |
| High grade | 53 | 4.33 (2.28-8.96) |  | 0.38 (0.19-0.51) |  |
| **Lymph node matastasis** | | | 0.62 |  | 0.27 |
| Negative | 96 | 4.30 (1.28-7.88) |  | 0.39 (0.20-0.63) |  |
| Positive | 14 | 4.45 (2.38-9.43) |  | 0.32 (0.22-0.43) |  |

**Supplementary Table S2: Correlation between urine lncRNA concentrations and clinicopathological characteristics of patients with BC in validation set [median (interquartile range)].**
