# Supplementary material for: Cell‐free lncRNA expression signatures in urine serve as novel non‐invasive biomarkers for diagnosis and recurrence prediction of bladder cancer
Source: J Cell Mol Med. 2018 Mar 8;22(5):2838–45. doi: 10.1111/jcmm.13578 (PMC5908122; doi:10.1111/jcmm.13578)
Supplement: Supplementary file 3 [file JCMM-22-2838-s003.docx]

| **Parameters** | **Categories** | **Univariate analysis** | |
| --- | --- | --- | --- |
|  |  | **HR (95% CI)** | ***p*-Value** |
| uc004cox.4 | Low vs. High | 0.598 (0.285-1.255) | 0.174 |
| GAS5 | Low vs. High | 0.907 (0.435-1.891) | 0.794 |

**Supplementary Table S3: Univariate Cox proportional hazards regression model analysis of recurrence-free survival in MIBC patients in validation set.**

Abbreviations: HR, hazard ratio; CI, confidence interval.
